# Self-assembly of robust gold nanoparticle monolayer architectures for quantitative protein interaction analysis by LSPR spectroscopy

**DOI:** 10.1007/s00216-020-02551-6

**Published:** 2020-03-21

**Authors:** Julia Flesch, Marie Kappen, Christoph Drees, Changjiang You, Jacob Piehler

**Affiliations:** 1 Department of Biology/Chemistry, University of Osnabrück, Barbarastr. 11, 49076 Osnabrück, Germany; 2 Center for Cellular Nanoanalytics (CellNanOs), University of Osnabrück, Barbarastr. 11, 49076 Osnabrück, Germany

**Keywords:** Localized surface plasmon resonance (LSPR), Self-assembly, Real-time biosensor, Protein immobilization, Quantitative interaction analysis, Kinetics

## Abstract

Localized surface plasmon resonance (LSPR) detection offers highly sensitive label-free detection of biomolecular interactions. Simple and robust surface architectures compatible with real-time detection in a flow-through system are required for broad application in quantitative interaction analysis. Here, we established self-assembly of a functionalized gold nanoparticle (AuNP) monolayer on a glass substrate for stable, yet reversible immobilization of Histidine-tagged proteins. To this end, one-step coating of glass substrates with poly-*L*-lysine graft poly(ethylene glycol) functionalized with ortho-pyridyl disulfide (PLL-PEG-OPSS) was employed as a reactive, yet biocompatible monolayer to self-assemble AuNP into a LSPR active monolayer. Site-specific, reversible immobilization of His-tagged proteins was accomplished by coating the AuNP monolayer with tris-nitrilotriacetic acid (trisNTA) PEG disulfide. LSPR spectroscopy detection of protein binding on these biocompatible functionalized AuNP monolayers confirms high stability under various harsh analytical conditions. These features were successfully employed to demonstrate unbiased kinetic analysis of cytokine-receptor interactions.

**Electronic supplementary material:**

The online version of this article (10.1007/s00216-020-02551-6) contains supplementary material, which is available to authorized users.

**Figure:**
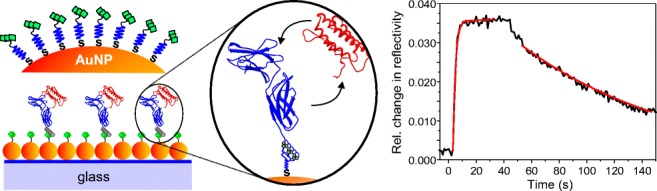
Graphical abstract

## Introduction

The highly dynamic and functional organization of biomolecules in cells is achieved by an extensive network of interactions that are intricately regulated in time and space. Understanding and describing cellular functions and their dysregulation at a systemic level therefore requires tools that enable large-scale quantification of kinetic and equilibrium constants of biomolecular interactions. Surface-based real-time monitoring by label-free detection offers elegant means for highly multiplexed interaction assays. Localized surface plasmon resonance (LSPR) spectroscopy has emerged as a highly sensitive, robust, and simple technique for label-free detection of biomolecular interactions [1–5]. Based on the collective electronic oscillation of metal nanoparticles (NP), LSPR spectroscopy probes the changes in refractive index highly confined to the NP surface. With its very high sensitivity and ease-of-use, LSPR spectroscopy has been highly successfully applied for label-free detection of protein–protein interactions in complex sample matrices and clinically relevant conditions [6–10]. For such applications, reliable analyses rely on maintenance of the colloidal stability of plasmonic nanoactuators and stable protein immobilization on the nanoplasmonic centers. This is even more critical for LSPR detection using clusters of noble metal nanoparticles, where coupling of electronic oscillations among the adjacent nanoparticles generates plasmonic “hotspots” [11, 12]. With a more than 10^6^-fold enhancement of the electromagnetic field, plasmonic hotspots have emerged as a cornerstone of a wide range of applications for surface-enhanced spectroscopies [13–15]. For instance, sensitivity down to the single molecule level has been achieved by surface-enhanced Raman spectroscopy [16, 17]. The electromagnetic properties of plasmonic hotspots are determined by material, shape, and spatial arrangement of metal nanoparticles [18–20].

For quantitative kinetic analysis, solid phase–based LSPR spectroscopy under flow-through conditions is desired, which requires a stable assembly of metal nanoparticles onto a solid substrate to ensure hotspot formation for reliable detection with highest sensitivity. Depositing metal nanoparticles on glass substrates exploiting electrostatic interactions is a commonly used method to obtain LSPR active layers on solid support [2, 3, 20]. Nanoparticle assemblies formed under these conditions, however, are susceptible to high ionic strength and pH changes. Moreover, further surface functionalization of non-covalently immobilized AuNP may destabilize the monolayer. Specifically, functionalization with thiols, which is most powerful for generating biocompatible surface coatings on AuNP or nanorods [4, 5, 7], efficiently competes with electrostatic interactions and therefore may remove nanoparticles from the support.

**Fig. 1:**
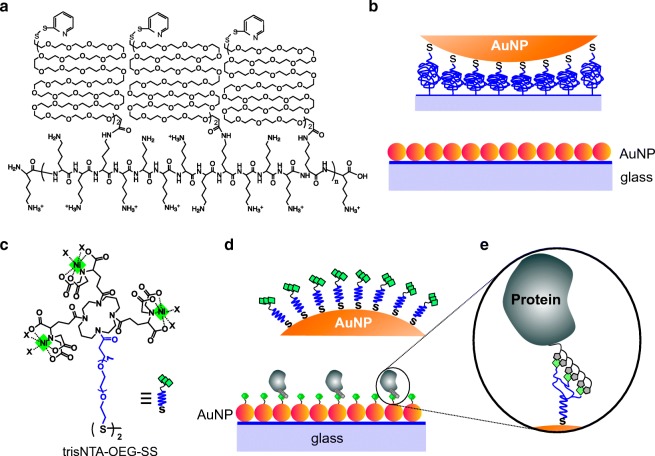
Self-assembly of functionalized AuNP monolayers for LSPR detection of protein interactions. **a** Molecular structure of PLL-PEG-OPSS for coating the substrate surface. **b** Schematic illustration of an AuNP monolayer on a PLL-PEG-OPSS-coated glass slide. **c** Molecular structure of trisNTA-OEG-SS with bound Ni^2+^ ions for reversible immobilization of His-tagged proteins. **d** Surface functionalization of AuNP monolayers for site-specific protein immobilization via functional thiols. **e** Scheme of multivalent hexahistidine-tagged protein binding on a trisNTA-OEG-SS-functionalized AuNP

Here, we developed a simple and robust approach to generate functionalized AuNP monolayers on glass substrates suitable for quantitative protein interaction analysis by LSPR spectroscopy with high sensitivity. For this purpose, we synthesized poly-*L*-lysine graft poly(ethylene glycol) terminated with ortho-pyridyl disulfide (PLL-PEG-OPSS) for surface functionalization of glass substrates, yielding a biocompatible, protein-repellent coating for selective AuNP deposition via Au-thiol interactions (Fig. 1a, b). Immobilized AuNP in turn were coated with disulfides comprising an OEG chain group and a functional group for site-specific protein capturing (Fig. 1c–e). Thus, we successfully implemented reversible immobilization of His-tagged proteins and demonstrate kinetic interaction analysis of cytokine-receptor interactions by LSPR spectroscopy.

## Experimental section

### Materials

Poly-*L*-lysine (PLL) hydrobromide with a molecular mass of 15–30 kDa was purchased from Sigma-Aldrich. Heterobifunctional poly(ethylene glycol) (3000 Da) with N-Hydroxysuccinimide ester (NHS) and ortho-pyridyl disulfide termini (NHS-PEG3k-OPSS) was purchased from Rapp Polymere GmbH, Tuebingen, Germany. TrisNTA-OEG7-disulfide was synthesized as described earlier [21]. OEG7 succinimidyl propionate disulfide (NHS-OEG7-SS) was purchased from Polypure, Norway. Gold nanoparticles (AuNP) with average diameter of 40 nm and citric acid coating were purchased from British Biocell International. All other chemicals were purchased from Sigma-Aldrich.

### Synthesis of PLL-PEG-OPSS

For synthesis of poly-L-lysine graft poly(ethylene glycol) terminated with ortho-pyridyl disulfide (OPSS), 30 mg OPSS-PEG3k-NHS (M 3073 Da), 7.5 mg poly-*L*-lysine hydrobromide (M 15–30 kDa), and 8 mg of N-(3-Dimethylaminopropyl)-N′-ethylcarbodiimide hydrochloride (EDC, M 192 Da) were dissolved in 400 μL HEPES buffer (100 mM HEPES, pH 7.5). After stirring for 8 h at room temperature, the mixture solution was dialyzed against ultrapure water (MilliQ, Merck) for 24 h. The sample was lyophilized, yielding 26 mg white powder stored at − 20 °C. The graft-modified polymer thus obtained was termed “PLL-PEG-OPSS.”

### Protein expression and purification

Monomeric enhanced green fluorescent protein carrying an N-terminal hexahistidine tag (H6-mEGFP) was expressed in *Escherichia coli* BL21 (DE3) and purified by immobilized metal ion affinity chromatography and size exclusion chromatography (Superdex 200 16/60, GE Healthcare) in a FPLC system (Äkta Explorer, GE Healthcare) [22]. mEGFP without oligohistidine tag was expressed in *E. coli*, purified by anion exchange column and size exclusion chromatography. The HaloTag fused to the anti-GPF nanobody “enhancer” [23] with an N-terminal decahistidine tag (H10-HaloTag-NB) was expressed in *E. coli* Rosetta (Novagen), purified by immobilized metal ion affinity and size exclusion chromatography as described previously [24].Wild-type IFNα2, the extracellular domain of IFNAR2 fused to a C-terminal decahistidine tag (IFNAR2-H10) and maltose binding protein fused to C-terminal decahistidine-tagged (MBP-H10), were expressed in *E. coli*, refolded from inclusion bodies, and purified as described before [25–27].

### Self-assembly and functionalization of AuNP monolayers

A suspension of 40 nm AuNP at OD 1 was concentrated in a SpeedVac to 1/6 of the initial volume (AuNPx6). Two 1 × 1 cm^2^ × 1 mm glass slides were cleaned for 10 min in air plasma to yield a hydrophilic negatively charged surface. 2 mg PLL-PEG-OPSS were dissolved in 1 mL HBS-20 buffer of pH 7.5 (20 mM HEPES, 150 mM NaCl). 1.5 mg TCEP were dissolved in 100 μL HBS-100 buffer of pH 7.5 (100 mM HEPES, 150 mM NaCl). 6 μL of the PLL-PEG-OPSS solution were sandwiched between both glass slides and PLL-PEG-OPSS was immobilized electrostatically to the negatively charged glass surface by the positive charges of its lysine backbone. After incubation for 10 min, the glass slides were separated; excess polymer was washed off with ultrapure water (milliQ, Merck) and dried with nitrogen. 8 μL TCEP solution were sandwiched between two PLL-PEG-OPSS-functionalized glass slides to reduce the disulfides of OPSS to thiols to enhance subsequent AuNP immobilization. After 10 min of incubation, the glass slides were separated, excess reducing agent was washed off with ultrapure water and dried with nitrogen. 30 μL of the AuNPx6 suspension were placed on each reduced PLL-PEG-OPSS-coated glass slide. After incubation for 45 min, the AuNPx6 drop was removed and the slides were immediately immersed in ultrapure water to prevent agglomeration of the formed AuNP monolayer. Glass slides coated with OPSS-PEG-PLL and AuNP are referred to as LSPR chips in the following and can be stored in ultrapure water at 4 °C for several weeks.

For further bioactive functionalization, LSPR chips were incubated with 0.3 mg/mL (0.1 mM) tris-(nitrilotriacetic acid)-OEG7-disulfide (trisNTA-OEG-SS) in HBS solution for 4 h, yielding a functional surface for probing reversible binding of His-tagged proteins. Functionalized LSPR chips were rinsed in ultrapure water and stored in ultrapure water at 4 °C.

### Reflectance spectroscopy

Protein immobilization and protein–protein interactions on immobilized AuNP monolayers were monitored in real time by LSPR reflectance spectroscopy as described previously [2], using a home-built setup previously established for label-free detection by reflectance interference spectroscopy (RIfS) [28–30]. A halogen light source is used to illuminate the LSPR chip mounted in a flow cell chamber via a bifurcated optical fiber. Reflected light is collected into the same optical fiber and recorded by a diode array spectrometer (see Electronic Supplementary Material (ESM) Fig. S1). Measurements were performed under continuous laminar flow-through conditions at 25 °C. A flow cell with a volume of ~ 200 nl was employed with typical flow rates of 200–500 μL/min, corresponding to flow velocities 200–500 cm/min. For LSPR detection, 1 mm glass slides were employed as substrates. For RIfS detection, the same setup was employed and glass substrates coated with a 325–400 nm silica layer were used as transducers [28]. In RIfS, a change in surface loading by 1 pg/mm^2^ leads to a shift of the interference minimum (1.5th order) by 1.2 pm as determined by calibration experiments with radioactively labeled proteins [31].

### LSPR data analysis

LSPR binding curves were obtained by monitoring the relative changes of the reflectivity over time. For this purpose, a reflectance spectrum was measured with a diode array detector from 450 to 700 nm for each time point. A MATLAB code was implemented to calculate binding curves from these spectral data. For every time point, the corresponding spectrum was interpolated to create smoother curves that translate into binding curves with a lower noise level. To correct for the underlying spectrum of the light source and the detector offset, a reference spectrum *S*_ref_ of an empty glass chip and offset *S*_0_ without light source, respectively, were measured. Raw spectral data *S*_raw_were corrected to *S*_corr_ using the following equation:

1 $$ {S}_{corr}=\frac{S_{raw}-{S}_0}{S_{ref}-{S}_0} $$

The calculation of the corresponding corrected spectrum from a raw spectrum is illustrated in Fig. S1b, c (see ESM). The averaged intensity from 20 data points around the LSPR peak maximum of each corrected reflectance spectrum was plotted as a function of time (EMS Fig. S1c, d). Subtraction of the initial intensity value at time *t* = 0 s yielded the binding curve in terms of time-dependent relative changes in reflectivity (ESM Fig. S1d). For the protein binding curves obtained on the functionalized AuNP monolayer, association and dissociation rate constants were quantified by fittings using the BIA evaluation 3.1 software (GE Healthcare). A standard kinetic model assuming a 1:1 Langmuir interaction as provided by the software was applied.

### Atomic force microscopy

Atomic fore microscopy was performed with a NanoWizard II AFM (JPK Instruments/Bruker). An AuNP monolayer was assembled on a 24 mm round glass coverslip as described above and immersed in ultrapure water. The height profile of the sample was measured in solution in intermitted contact mode using a silicon tip from nanosensors with a resonance frequency of 204–497 kHz and a force constant of 10–130 N/m.

## Results and discussion

### Formation of self-assembled AuNP monolayer on glass

PLL-PEG derivatives offer versatile means for functionalization of glass substrates [24, 32, 33]. They spontaneously adhere to glass-type surfaces at neutral pH by multivalent electrostatic interactions of positively charged PLL amine groups with the negatively charged silanol groups. PLL-PEG-coated glass substrates exhibit high stability at physiological pH and are therefore well compatible with biological applications including cell culture conditions [34, 35]. Here, we synthesized poly-*L*-lysine graft poly(ethylene glycol) terminated with ortho-pyridyl disulfide (PLL-PEG-OPSS) (Fig. 1a) to generate a biocompatible, protein-repellent surface coating for self-assembly of AuNP monolayers (Fig. 1b). By controlling the ratio of PEGs graft to the lysine moieties of PLL, 30% of the ε-amine moieties in poly-*L*-lysine are conjugated with PEG-OPSS and the remaining are left unmodified for electrostatic interaction. Such PEG-to-lysine ratio yields high biocompatibility while maintaining sufficient amine density to warrant stable adsorption onto glass substrates [36–38].

**Fig. 2:**
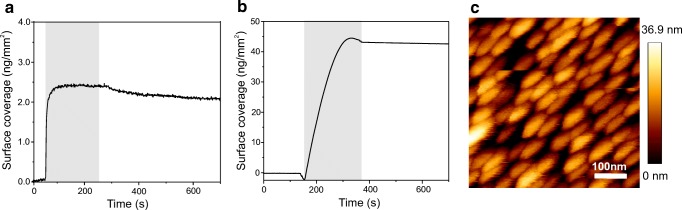
Self-assembly of 40 nm AuNP monolayers on PLL-PEG-OPSS-coated surfaces. **a** Formation of a stable PLL-PEG-OPSS polymer coating on a silica substrate monitored by reflectance interference spectroscopy (RIfS). **b** Real-time RIfS binding curve of 6-fold concentrated AuNPs to PLL-PEG-OPSS-coated silica substrate. **c** AFM image of the AuNP monolayer in buffer solution

Coating of glass-type surfaces with PLL-PEG-OPSS and subsequent assembly of AuNP was monitored by reflectance interference spectroscopy (RIfS) in a flow-through system. Rapid binding of PLL-PEG-OPSS with a saturation mass change of ~ 2 ng/mm^2^ was observed (Fig. 2a), which is in good agreement to the formation of a PLL-PEG brush as reported previously [2, 24]. Minor dissociation was observed upon washing with buffer, confirming formation of a stable PLL-PEG-OPSS monolayer on the silica surface of the RIfS transducer. Further on, a face-to-face sandwich method as described in the “Experimental section” was used for coating substrates with PLL-PEG-OPSS in order to minimize sample consumption. Binding of 40 nm AuNPs to PLL-PEG-OPSS-coated surfaces was likewise monitored in real time by RIfS. To accelerate the AuNP layer formation on PLL-PEG-OPSS-coated glass, the OPSS disulfide bond was reduced to thiol groups by incubation with tris(2-carboxyethyl)phosphine (TCEP) prior to the injection of AuNPs. Under these conditions, rapid binding of AuNP to the activated surface was detected upon injection of AuNP solution (Fig. 2b). The linear slope observed for the initial phase of the binding curve suggests diffusion-controlled AuNP binding to the PLL-PEG-SH-functionalized surface.

Based on these results, self-assembled AuNP films on glass slides were prepared from AuNP solutions with 6- and 10-fold concentrations, respectively. Glass slides obtained from 6- and 10-fold concentrations (AuNPx6 and AuNPx10) yielded dark purple layers on the glass slides in line with formation of AuNP monolayers. They were used as LSPR chips to survey optimized conditions in the following LSPR spectroscopy detections. Atomic force microscopy (AFM) images of an AuNP monolayer prepared from a AuNPx6 solution confirmed dense packing with AuNPs contacting each other (Fig. 2c). Absence of nanoparticle aggregations and multilayer stacks confirmed the formation of a self-assembled AuNP monolayer.

### LSPR detection and sensitivity to changes in the bulk refractive index

**Fig. 3:**
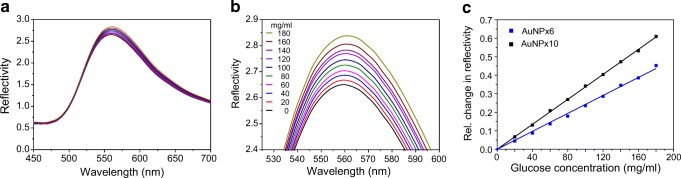
Responsiveness of AuNP monolayers quantified by LSPR reflectance spectroscopy. **a**, **b** Full reflectance spectra **a** and zoom into the peak reflectivity **b** of an AuNP monolayer assembled on a PLL-PEG-OPSS-coated glass slide upon injecting glucose at different concentrations. Glucose concentrations are indicated in the legend of panel B. **c** Linear correlation of the relative reflectivity determined at the peak of the reflectance spectra with the glucose concentration. The relative reflectivity was determined by subtracting the reflectivity in the absence of glucose. LSPR chips were prepared from 6- (blue) or 10-fold (black) concentrated AuNP solutions

These dense AuNP monolayers were employed for LSPR detection by white light reflectometry. For this purpose, AuNP-coated glass substrates were mounted into a flow cell connected via fiber optics with a tungsten halogen lamp and a diode array spectrometer (ESM Fig. S1). Changes in the LSPR reflectivity of the AuNP monolayer upon changes in the bulk refractive index were explored by injecting glucose solution at various concentrations. Reflectance spectra of LSPR chips coated with different AuNP densities (AuNPx6, AuNPx10) were compared. The glucose experiments were used to quantify the sensitivity of the LSPR signal in response to changes of the refractive index (Fig. 3a). For AuNPx6-coated LSPR chips, a significant increase in the reflectance amplitude was observed upon exposition to higher concentrations of glucose (Fig. 3b). A linear correlation between the increase of the reflectivity at the LSPR peak and the refractive index in glucose solution was obtained (Fig. 3c). Only minor changes in the position of the reflectance maximum on the wavelength axis could be detected, and the signal-to-noise ratio was much higher for the shift in intensity (ESM Fig. S2). Therefore, subsequent LSPR detection was based on monitoring changes in intensity over time (s. “Experimental section”). For the 100 mg/mL glucose solution, which corresponds to a change in refractive index of 0.0147, a relative change in reflectivity Δ*R* = 0.23 was observed, yielding a bulk refractive index sensitivity of Δ*R* = 15.6 per refractive index unit. With a root-mean-square noise (RMSE) of 6.5 × 10^−4^ in this experiment, a signal-to-noise of ~ 24,000 per refractive index unit is achieved. A sensitivity of Δ*R* = 23.1 per refractive index unit was observed for the AuNPx10-coated LSPR chips (Fig. 3c). For practical reasons related to the coating procedure and for avoiding saturation of the detection system, however, all further experiments were carried out with AuNPx6-coated LSPR chips.

### Functionalization of AuNP monolayers for site-specific protein immobilization

In order to obtain site-specific protein immobilization for quantitative protein interaction analysis, we implemented surface modification of AuNP monolayers by functionalized thiols (cf. Fig. 1c–e). For this purpose, LSPR chips were incubated with 0.3 mg/mL of tris-(nitrilotriacetic acid)-OEG7-disulfide (trisNTA-OEG-SS) in HBS solution. TrisNTA is a multivalent chelator that binds oligohistidine-tagged (His-tagged) proteins via complexed transition metal ions (e.g., Ni^2+^) in a stable yet reversible manner, which has been successfully applied for functional protein immobilization on various substrate materials [39–41] (Fig. 1c). To obtain a dense trisNTA functionalization on AuNP, the LSPR chips were incubated in trisNTA-OEG-SS solution for 4 h. No significant change in the color was observed after this treatment, indicating that aggregation of immobilized AuNP does not occur under these conditions.

**Fig. 4:**
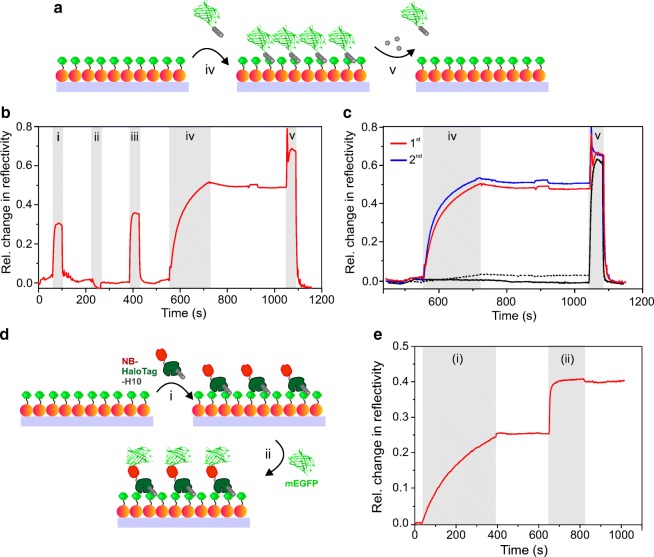
Specific and reversible immobilization of His-tagged proteins onto AuNP monolayers functionalized with trisNTA-OEG-SS. **a** Cartoon depicting reversible immobilization of H6-mEGFP. **b** Typical binding assay detected by LSPR spectroscopy including the following sample injections: (i) 250 mM EDTA, (ii) 10 mM NiCl_2_, (iii) 500 mM imidazole, (iv) 1 μM H6-mEGFP, and (v) 500 mM imidazole. **c** Immobilization of H6-mEGFP repeated twice on the same LSPR chip and control experiments using mEGFP without His-tag (dashed black line), or H6-mEGFP without Ni^2+^ ions (black line). **d** Scheme of capturing tagless mEGFP via H10-HaloTag-NB immobilized on a trisNTA-functionalized LSPR chip. **e** On the Ni^2+^-ion conditioned AuNP monolayer, injections are (i) 300 nM H10-HaloTag-NB, and (ii) 100 nM of tagless mEGFP

Functionalizing the AuNP monolayer by trisNTA-OEG-SS paves the way for site-specific immobilization of His-tagged proteins on LSPR chips. We first explored binding of hexahistidine-tagged monomeric enhanced green fluorescence protein (H6-mEGFP) onto trisNTA-functionalized LSPR chips. After loading Ni^2+^ ions, characteristic binding of H6-mEGFP to the AuNP surface was observed as detected by an increase in reflectivity (Fig. 4b). During washing with buffer, the protein remained stably bound to the surface until an injection of 500 mM imidazole as competitor for Histidine, which completely removed the protein from the surface (Fig. 4b). A subsequent immobilization cycle yielded a very similar level of mEGFP on the surface demonstrating reproducible site-specific protein immobilization on the trisNTA-functionalized LSPR chip binding (Fig. 4c). Specificity of His-tag mediated immobilization was furthermore confirmed by repeating the same experiment with tagless mEGFP, which yielded negligible protein binding (Fig. 4c). Likewise, no binding of mEGFP was observed in the absence of Ni^2+^ ions (Fig. 4c). His-tagged protein binding and robust changes of LSPR reflectivity were observed for repeating the injection twice on the same surface (Fig. 4c).

We furthermore explored site-specific capturing of GFP via an immobilized anti-GFP nanobody (NB). To this end, NB fused to HaloTag with a decahistidine tag (H10-HaloTag-NB) was immobilized on a trisNTA-functionalized LSPR chip (Fig. 4d, e). Rapid and stable binding of mEGFP to the H10-HaloTag-NB was observed as expected for their very high interaction affinity [38]. The ratio of the LSPR signals of mEGFP to H10-HaloTag-NB was 0.53. This value is in excellent agreement to the ratio of the molecular masses of mEGFP and H10-HaloTag-NB, i.e., 26.9 kDa/47.7 kDa = 0.56. These results confirmed full functional integrity of the immobilized NB, providing the capability for efficient capturing of GFP-tagged proteins onto trisNTA-functionalized LSPR chips.

The stability of the functionalized AuNP monolayer for protein immobilization and interaction was explored by repeated immobilization cycles. Highly consistent binding capacity and kinetics were observed on the same LSPR chip after 6 times repeating of H6-mEGFP injections and imidazole washes (ESM Fig. S4a). Furthermore, the stability of the self-assembled AuNP monolayer at low pH, high ionic strength, and reduction conditions was assessed. For this purpose, 100 mM HCl, 1 M NaCl, and 500 mM dithiothreitol (DTT), respectively, were injected to the same LSPR chip, each followed by immobilization of H6-mEGFP. Largely identical binding curves were observed for H6-mEGFP after these treatments (ESM Fig. S4b), confirming an excellent robustness of the LSPR chip for protein interaction analysis under different environments.

### Reversible protein binding and quantitative protein interaction analysis

**Fig. 5:**
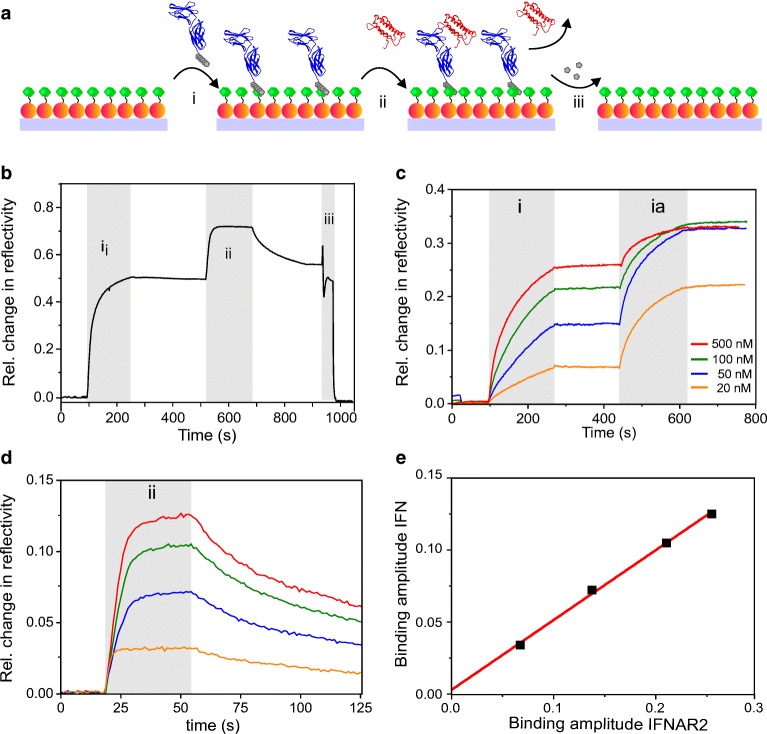
Reversible protein–protein interaction analysis by LSPR reflectance spectroscopy. **a** Cartoon of the assay. Immobilization of IFNAR2-H10 (blue, i), reversible binding of IFNα2 (red, ii) and surface regeneration by imidazole (iii) on a trisNTA-functionalized AuNP monolayer. **b** Changes in reflectivity upon injection of 500 nM IFNAR2-H10 (i), 500 nM IFNα2 (ii), and 500 mM imidazole (iii). **c**–**e** Dependency of ligand binding to receptor densities quantified by LSPR reflectance spectroscopy. **c** Alternation of receptor surface densities on a trisNTA-functionalized AuNP monolayer by injecting different concentrations of IFNAR2-H10 (i), followed by an injection of 1 μM MBP-H10 (ia). **d** LSPR signals of binding 500 nM IFNα2 onto immobilized IFNAR2-H10 with different densities. Color coding of curves is the same as in panel **c**. **e** Plots of relative LSPR reflectivity amplitudes of bound IFNα2 versus immobilized IFNAR2-H10. The red line shows linear regression yielding a slope of 0.49 ± 0.02 and an intercept 0.003 ± 0.0001

To explore whether the functionality of representative, biomedically relevant proteins was preserved upon immobilization on trisNTA LSPR chips, we immobilized the ectodomain of the type I interferon receptor subunit 2 fused to a C-terminal decahistidine tag (IFNAR2-H10). After immobilization of IFNAR2-H10, the interaction with the ligand interferon-α2 (IFNα2) was probed (Fig. 5a). Rapid association of IFNα2 to the immobilized IFNAR2-H10 was observed during injection, followed by dissociation during washing with buffer as expected for this reversible protein−protein interaction [25] (Fig. 5b). The obtained LSPR signal as the relative change in reflectivity is 0.48 for a saturated IFNAR2-H10 binding. The corresponding root-mean-square error (RMSE) was determined as 6.05 × 10^−4^ (ESM Fig. S3). Given the mass of ~ 5 ng/mm^2^ for an IFNAR2 monolayer as found previously on various densely functionalized planar surface architectures [2, 25, 42], a detection limit of ~ 10 pg/mm^2^ for protein binding was estimated. The sensitivity of ~ 0.1 relative change in reflectivity per ng/mm^2^ protein was similar as previously reported for related AuNP surface architectures [2].

At these surface-saturating conditions, binding of IFNα2 was clearly biased by mass transport limitations as evident from the dissociation curve. We therefore probed binding at different surface densities of IFNAR2-H10. To this end, IFNAR2-H10 was injected at concentrations of 20 nM, 50 nM, 100 nM, and 500 nM, respectively, on the trisNTA-functionalized AuNP monolayer. Remaining trisNTA immobilization sites were blocked by excess decahistidine-tagged maltose binding protein (MBP-H10) to minimize non-specific interactions (Fig. 5c). IFNα2 with a constant concentration of 500 nM was injected after blocking with MBP-H10 to ensure saturated binding to IFNAR2 (*K*_D_ ~ 10 nM) (Fig. 5d). The LSPR signals of the bound IFNα2 versus immobilized IFNAR2-H10 at different concentrations were plotted (Fig. 5e). The LSPR signal amplitude observed for IFNα2 linearly increased with IFNAR2-H10 surface loading (*R*^2^ 0.9935). The fit intercept of 0.003 confirmed a high binding specificity of IFNα2 to the immobilized IFNAR2-H10. The slope of 0.49 ± 0.02 significantly falls below the ratio of the molecular mass of IFNα2 and IFNAR2-H10 (18.2 kDa/26.0 kDa = 0.7). The reason could be a non-linear dependency of refractive index to molecular mass, different sensitivities caused by different distances from the AuNP surface [43] or partially inactive IFNAR2-H10. However, these measurements clearly established robust capability for detecting ligand-receptor interactions.

**Fig. 6:**
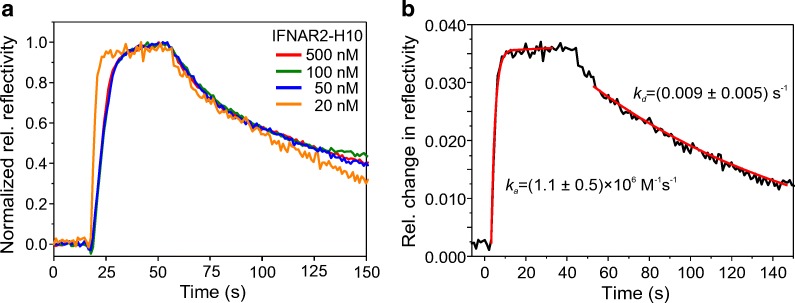
Quantification of the IFNα2-IFNAR2 interaction kinetics by LSPR reflectance spectroscopy. **a** Normalized IFNα2 binding curves obtained for different densities of immobilized IFNAR2-H10. Concentrations of IFNAR2-H10 used for immobilization are indicated by the legend. **b** Fitting of association and dissociation phases (red lines), respectively, of the IFNα2 binding curve obtained upon immobilization of IFNAR2-H10 at 20 nM

For comparing the IFNα2 binding kinetics at different IFNAR2-H10 surface densities, we normalized the binding curves of IFNα2 to surface-immobilized IFNAR2-H10 obtained from concentrations of 20 nM, 50 nM, 100 nM, and 500 nM, respectively (Fig. 6a). Significantly faster association and dissociation of IFNα2 was observed for experiments obtained at 20 nM IFNAR2-H10, which is in line with mass transport-limited binding at elevated IFNAR2-H10 surface densities. At the lowest IFNAR2-H10 density, largely unbiased association and dissociation kinetics of the IFNα2-IFNAR2 interaction were observed in repeated binding experiments (ESM Fig. S5), which could be fitted by a monoexponential Langmuir model (Fig. 6b). Based on these binding curves, an association rate constant of (1.1 ± 0.5) × 10^6^ M^−1^ s^−1^ and a dissociation rate constant of 0.009 ± 0.005 s^−1^ were obtained from the fit. These results are in excellent agreement with previous measurements of this interaction by solid-phase detection techniques [29]. These results confirmed functional protein immobilization into the AuNP surface architecture and unbiased interaction analysis.

## Conclusions

Self-assembled monolayers of thiol-containing compounds on Au are a robust method to introduce biocompatible surface functionalization of Au-based substrates for surface plasmon resonance (SPR) detection. In this work, we revised the concept for formation of self-assembled AuNP monolayer on a glass substrate to obtain a reliable protein interaction analysis by LSPR spectroscopy detection. Coating of glass surfaces with PLL-PEG-OPSS provides dense disulfide moieties that can be readily reduced to thiols. Thus, stable self-assembled AuNP monolayers are obtained by multiple Au-thiol interactions. Importantly, the PEG polymer brush assembled via the PLL-graft copolymer potently prevents non-specific protein adsorption onto the glass substrate and thus minimizes potential bias of LSPR detection in complex physiological sample matrices. Thiol-mediated assembly of the AuNP monolayers in turn enabled versatile biocompatible functionalization with thiol-containing compounds. This is in a stark contrast to AuNP monolayers formed by electrostatic interactions, where thiol-containing compounds induced aggregation of AuNPs and removal from the substrate [2]. For proof-of-concept experiments, we employed AuNP surface functionalization with a trisNTA-thiol for site-specific, reversible immobilization of His-tagged proteins. Thus, efficient and stable, yet reversible protein immobilization was achieved. Proteins immobilized into these surface architectures maintained their capability to recognize their interaction partners with uncompromised affinity and kinetics. TrisNTA-functionalized AuNP monolayers obtained in this work proved highly stable under very harsh conditions, such as 0.1 M HCl, 1 M NaCl, and 500 mM dithiothreitol. The high stability paves the way for applications of LSPR spectroscopic detection or imaging in live cells, as well as in protein interaction analysis at clinically relevant conditions. Moreover, the surface architecture established here is compatible with other LSPR active metal nanoparticles, such as silver or copper, and with alternative biocompatible functionalization, e.g., via biotinylated thiols or the HaloTag ligand [44], which has been successfully applied for cell surface capturing [36]. Such robust and versatile AuNP surface architectures open exciting possibilities for the application not only for quantitative interaction analysis by LSPR detection but also for vibrational spectroscopy on immobilized proteins by surface-enhanced Raman spectroscopy (SERS).

## Electronic supplementary material

ESM 1 (PDF 567 kb)

## Abbreviations

AuNP: Gold nanoparticle
HaloTag-NB: HaloTag fused with anti-GFP nanobody
HTL: HaloTag ligand
IFNAR2: Type I interferon receptor subunit 2
IFNα2: Interferon-α2
LSPR: Localized surface plasmon resonance
mEGFP: Monomeric enhanced green fluorescent protein
PLL-PEG-OPSS: Poly-*L*-lysine graft poly(ethylene glycol) terminated with ortho-pyridyl disulfide
TrisNTA: Tris-nitrilotriacetic acid
