# Supplementary material for: Self-assembly of robust gold nanoparticle monolayer architectures for quantitative protein interaction analysis by LSPR spectroscopy
Source: Anal Bioanal Chem. 2020 Mar 21;412(14):3413–22. doi: 10.1007/s00216-020-02551-6 (PMC7214499; doi:10.1007/s00216-020-02551-6)
Supplement: Supplementary file 1 — (PDF 567 kb) [file 216_2020_2551_MOESM1_ESM.pdf]

Analytical and Bioanalytical Chemistry

Electronic Supplementary Material

Self-assembly of robust gold nanoparticle monolayer architectures for quantitative protein interaction analysis by LSPR spectroscopy

Julia Flesch, Marie Kappen, Christoph Drees, Changjiang You, Jacob Piehler

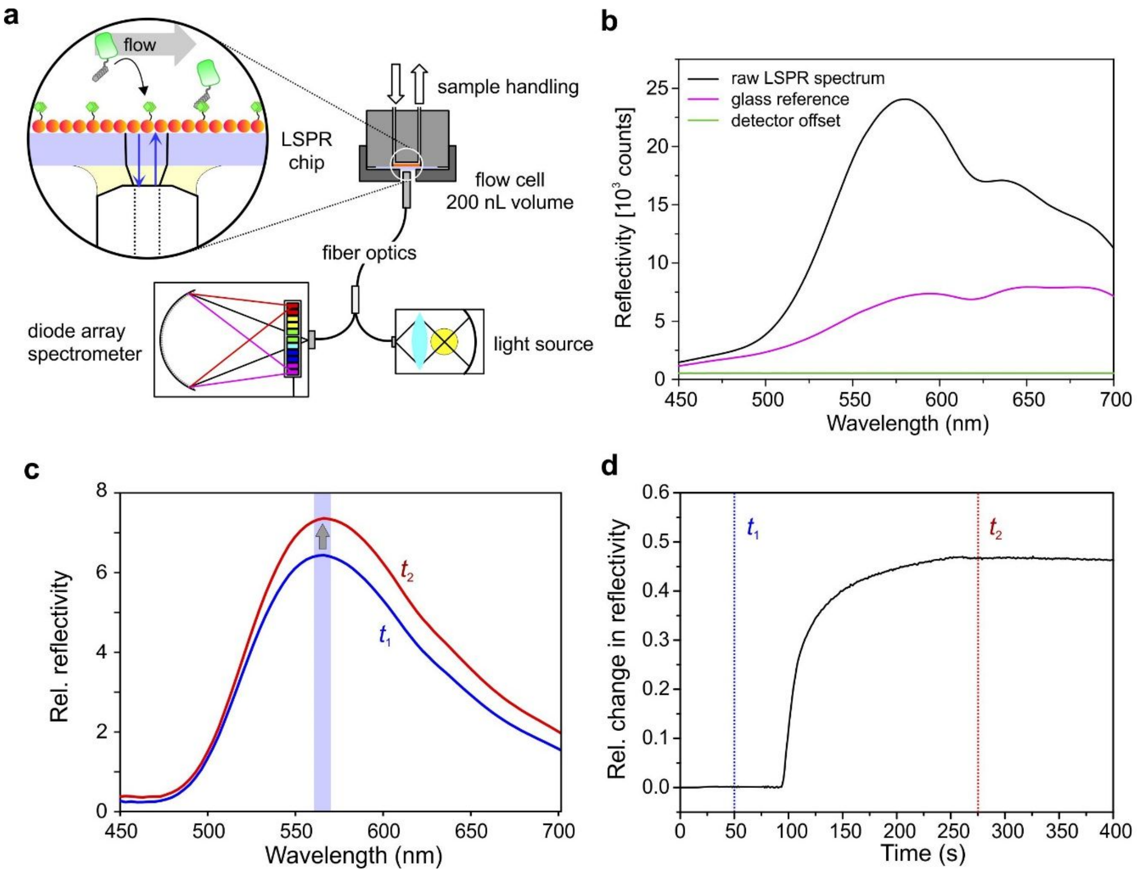

Fig. S1 Reflectometric LSPR spectroscopy. (a) Scheme of the setup for LSPR detection by white light reflectometry. (b) Measured raw LSPR reflection spectrum on AuNP monolayer (dark line), reference reflection spectrum of an empty glass chip (magenta) and detector offset without illumination (green) for obtaining the corrected spectrum using equation (1). (c) Corrected LSPR spectra shown as relative reflectivity vs wavelength. Data points of ±5 nm around the LSPR peak were averaged and plotted as a function of time (gray zone). LSPR spectra before (t_1, blue) and after (t_2, red) protein binding are shown. (d) Subtraction of the initial reflectivity at time $t = 0$ s yielded the binding curve in terms of relative changes in reflectivity. Dashed lines indicate the time points t_1 and t_2, respectively, corresponding to the spectra shown in panel c

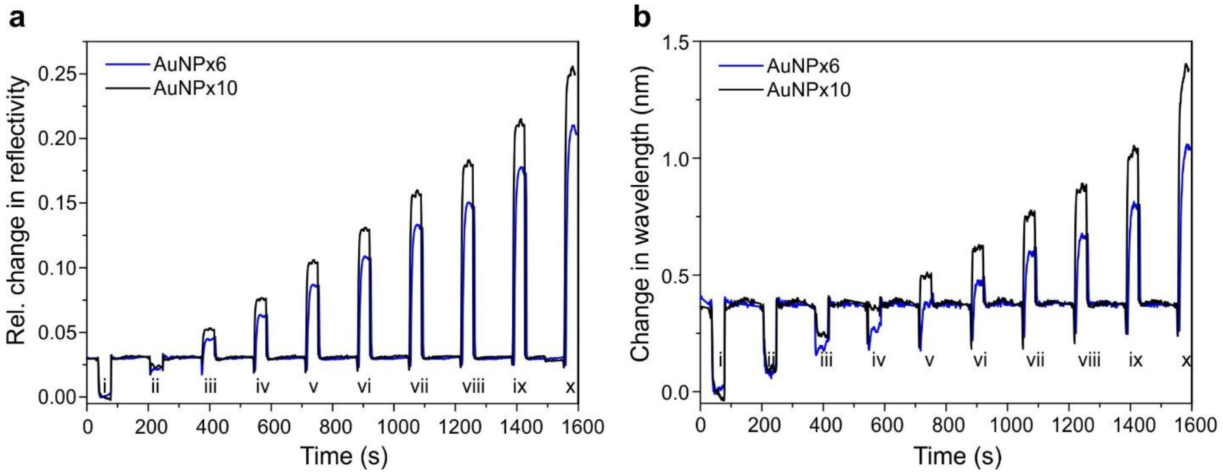

Fig. S2 Changes in intensity (a) and wavelength (b) of the LSPR reflection maximum upon bulk refractive index changes during injection of glucose in different concentrations (i: 0 mM; ii: 20 mM; iii: 40 mM; iv: 60 mM; v: 80 mM; vi: 100 mM; vii: 120 mM; viii: 140 mM; ix: 160 mM; x: 180 mM)

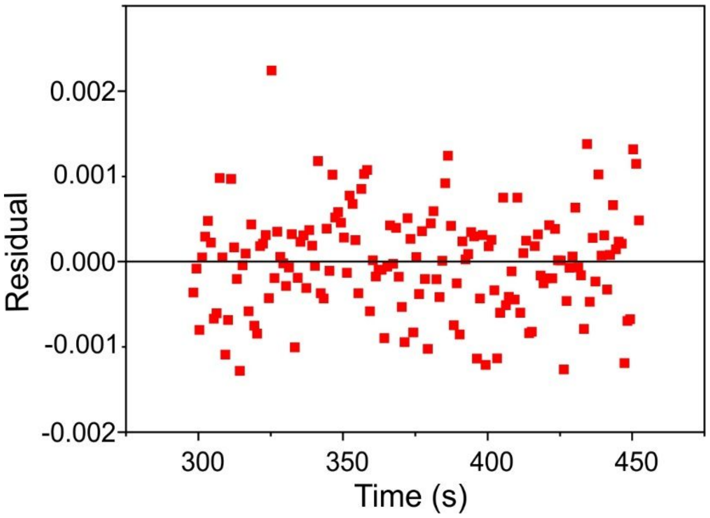

Fig. S3 Residuals from linear regression of the baseline for quantifying the noise level

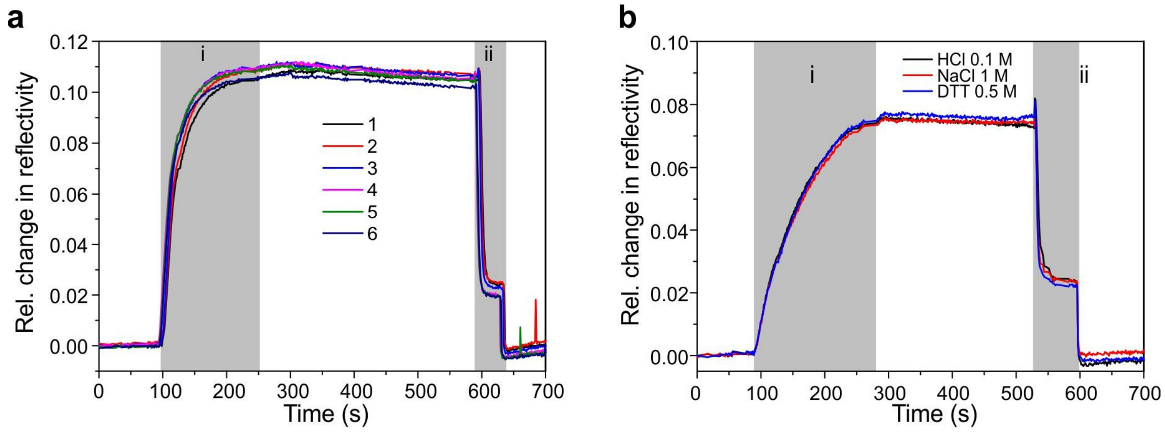

Fig. S4 Stability of trisNTA-functionalized AuNP monolayer self-assembled on PLL-PEG-OPSS-coated glass. (a) Repeated immobilization of 500 nM H6-mEGFP (i) on the same trisNTA-OEG-SS-functionalized AuNP monolayer pre-conditioned with Ni^{2+} ions and removal by 250 mM imidazole (ii). The number of the six consecutive experiments are indicated in the legend. (b) Immobilization of H6-mEGFP on AuNP monolayer after treatment with 0.1 M HCl, 1 M NaCl and 0.5 M dithiothreitol (DTT), respectively. The same injections (i) and (ii) as shown in the assays of panel a were carried out two days later on the same LSPR chip, which remained mounted in the flow cell

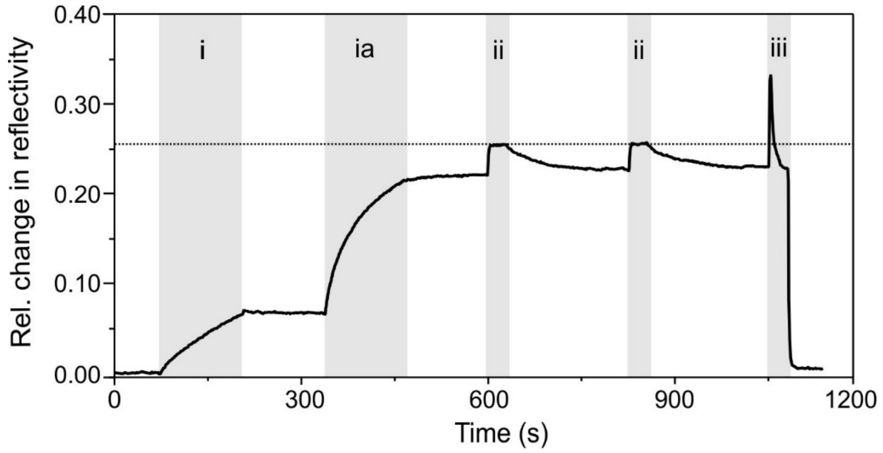

Fig. S5 Repeated binding of IFNα2 to IFNAR2-H10 immobilized on trisNTA-functionalized AuNP monolayer for determining binding kinetic constants. Injections on the Ni^{2+}- conditioned surface are: 20 nM IFNAR2-H10 (i), 1 µM MBP-H10 (ia), 500 nM IFNα2 twice (ii), and 500 mM imidazole (iii). The dashed line marks the saturated IFNα2 binding amplitude
